# CRITICAL ANALYSIS OF SURGICAL TREATMENT TECHNIQUES OF MORBID OBESITY

**DOI:** 10.1590/0102-672020190001e1450

**Published:** 2019-10-21

**Authors:** Bruno ZILBERSTEIN, Marco Aurélio SANTO, Marnay Helbo CARVALHO

**Affiliations:** 1 Department of Gastroenterology, School of Medicine, University of São Paulo, São Paulo, SP, Brazil

**Keywords:** Gastroplasty, Gastric bypass, Body mass index, Obesity, Bariatric surgery, Cirurgia bariátrica, Gastroplastia, Índice de massa corporal, Obesidade mórbida, Derivação gástrica

## Abstract

**Introduction:**

Obesity is a disease of high prevalence in Brazil and in the world, and bariatric surgery, with its different techniques, is an alternative treatment.

**Objective:**

To compare techniques: adjustable gastric band (AGB), sleeve gastrectomy), Roux-en-Y gastric bypass (RYGB) and biliopancreatic diversion (BPD) analyzing leaks, bleeding, death, weight loss, resolution of type 2 diabetes, systemic arterial hypertension, dyslipidemia and obstructive sleep apnea.

**Methods:**

Were selected studies in the PubMed database from 2003 to 2014 using the descriptors: obesity surgery; bariatric surgery; biliopancreatic diversion; sleeve gastrectomy; Roux-en-Y gastric bypass and adjustable gastric banding. Two hundred and forty-four articles were found with the search strategy of which there were selected 116 studies through the inclusion criteria.

**Results:**

Excess weight loss (EWL) after five years in AGB was 48.35%; 52.7% in SG; 71.04% in RYGB and 77.90% in BPD. The postoperative mortality was 0.05% in the AGB; 0.16% on SG; 0.60% in RYGB and 2.52% in BPD. The occurrence of leak was 0.68% for GBA; 1.93% for SG; 2.18% for RYGB and 5.23% for BPD. The incidence of bleeding was 0.44% in AGB; 1.29% in SG; 0.81% in RYGB and 2.09% in BPD. The rate of DM2 resolved was 46.80% in AGB, 79.38% in SG, 79.86% in RYGB and 90.78% in BPD. The rate of dyslipidemia, apnea and hypertension resolved showed no statistical differences between the techniques.

**Conclusion:**

The AGB has the lowest morbidity and mortality and it is the worst in EWL and resolution of type 2 diabetes. The SG has low morbidity and mortality, good resolution of comorbidities and EWL lower than in RYGB and BPD. The RYGB has higher morbidity and mortality than AGB, good resolution of comorbidities and EWL similar to BPD. The BPD is the worst in mortality and bleeding and better in EWL and resolution of comorbidities.

## RESUMO

**Introdução:**

A obesidade é afecção de alta prevalência no Brasil e no mundo e a cirurgia bariátrica, com suas diferentes técnicas, é alternativa para o tratamento.

**Objetivo:**

Comparar as técnicas da banda gástrica ajustável (BGA), gastrectomia vertical (GV), gastroplastia com derivação em Y-de-Roux (GDYR) e derivação biliopancreática (DBP) focando fístula, sangramento, óbito, perda e reganho ponderal, e resolução das comorbidades diabete melito tipo 2 (DM2), hipertensão arterial sistêmica (HAS), dislipidemia e apneia obstrutiva do sono (AOS).

**Métodos:**

Buscou-se os estudos na base de dados PubMed de 2003 a 2014 usando os descritores: obesity surgery; bariatric surgery; biliopancreatic diversion; sleeve gastrectomy; Roux-en-Y gastric bypass e adjustable gastric banding*.* Dessa busca foram recuperadas 244 publicações sendo selecionados 116 após aplicar os critérios de inclusão/exclusão.

**Resultados:**

A perda de excesso de peso (PEP) após cinco anos foi 48,35% na BGA; 52,7% na GV; 71,04% na GDYR e 77,90% na DBP. A mortalidade pós-operatória foi 0,05% na BGA; 0,16% na GV; 0,60% na GDYR e 2,52% na DBP. A ocorrência de fístulas foi 0,68% para BGA; 1,93% para GV; 2,18% para GDYR e 5,23% para DBP. A ocorrência de sangramento foi 0,44% na BGA; 1,29% na GV; 0,81% na GDYR e 2,09% na DBP. A taxa do DM2 resolvida foi de 46,80% na BGA, 79,38% na GV, 79,86% na GDYR e 90,78% na DBP. A taxa de dislipidemia, apneia e hipertensão resolvidas não demonstraram diferenças estatísticas entre as técnicas.

**Conclusões:**

A BGA apresenta a menor morbimortalidade e é a pior em PEP e resolução do DM2. A GV apresenta baixa morbimortalidade, boa resolução das comorbidades e PEP inferior às GDYR e DBP. A GDYR apresenta morbimortalidade superior à BGA, boa resolução das comorbidades e PEP semelhante à DBP. A DBP é a pior em mortalidade e sangramento e melhor em PEP e resolução das comorbidades.

## INTRODUCTION

According to the World Health Organization obesity is a condition of high prevalence in Brazil and in the world and it is on the rise^25^. It is defined as accumulation of fatty tissue, which leads to an increase in the body mass index (BMI) to 30 kg/m^2^ or more and it is considered a risk factor for type 2 diabetes mellitus (T2DM), systemic arterial hypertension (SAH) ), dyslipidemia and obstructive sleep apnea (OSA) among other diseases.

Because it is a chronic disease, in the treatment of obesity there must be patient compliance and follow-up with health professionals even after achieving the goal of ideal weight^5^ ^,^ ^23^. Currently, conservative treatments (performed through dietary reeducation measures and physical activity practices, with or without the use of medications) and surgical treatments are considered as therapeutic modalities. Although weight loss with conservative measures, only 5-10% of this population maintains the loss of excess weight in the long term^11^. In this sense, bariatric surgery may be indicated in patients with BMI greater than 40 kg/m^2^ or BMI greater than or equal to 35 kg/m^2^ associated with comorbidities^2^ ^,^ ^6^ ^,^ ^18^.

Today Brazil occupies the second position in the world regarding the accomplishment of bariatric procedures, which contributes to the development of the treatment of this global epidemic.

Therefore, this study proposes to analyze the techniques that have been described most in the literature, being the adjustable gastric band (AGB), vertical gastrectomy (VG), Roux-en-Y gastric bypass (RYGB) and biliopancreatic derivation (BPD) for complications and operative mortality, loss and overweight, and resolution of comorbidities (DM2, hypertension, dyslipidemia and OSA) with the intention of evaluating possible differences between them, thus contributing to an objective critical assessment regarding the choice of the best technique for each patient.

## METHODS

A literature review was made with reference to the cited and published techniques between 2003 and 2014. Were considered the prospective and retrospective studies written in the English language. The search was performed in PubMed using the descriptors: obesity surgery; bariatric surgery; biliopancreatic diversion; sleeve gastrectomy; Roux-en-Y gastric bypass and adjustable gastric banding from which 244 publications were retrieved; 128 were discarded after applying the inclusion/exclusion criteria and 116 studies containing 24,818 surgeries were selected for this analysis.

### Inclusion criteria

The AGB, SG, RYGB and BPD techniques were considered for statistical analysis and the last one containing both the “duodenal switch” and “Scopinaro”, since both are conceptually similar. In the selection of publications, both laparoscopic and laparotomy procedures were considered, which presented results regarding postoperative morbidity and mortality in the first 30 days (leaks, bleeding and death); loss and regain of overweight, and resolution of comorbid DM2, SAH, dyslipidemia and OSA.

### Exclusion criteria:

Works done in animals, with small cases (less than 10 patients), follow-up of less than 30 days, and reports of cases/citations were excluded.

The studied aspects were: the preoperative characteristics of the patients; complications and postoperative mortality in the first 30 days (leaks, bleeding and death); loss and regain of excess weight; resolution of comorbidities (DM2, SAH, dyslipidemia and OSA) - being considered as resolution those patients who no longer needed medications and/or had normal values after the operation.

### Statistical analysis

Statistical Analysis was performed using the R environment: “A Language and Environment for Statistical Computing (2014)”. In order to compare the four groups (AGB, SG, RYGB and BPD), a variance analysis (ANOVA) of a completely randomized design (DIC) was performed on each of the variables analyzed. Once the significance of the F test was determined, the Duncan test was performed for multiple comparison of the averages of the variables (DM2, SAH, dyslipidemia and AOS and EWL)^1^. In the case of variables that did not meet the assumptions of normality, independence of the residues and the homogeneity of the variances, the logarithmic transformation was performed and the analysis of variance was performed with the multiple comparison test. The non-parametric Kruskal-Wallis test was applied to the variables that did not solve the presupposition problem (age, BMI, gender, bleeding, leaks and death). For each group, it was verified whether the data followed normal distribution by the Shapiro-Wilk test (p>0.05). Interval estimation of the sample averages was also performed from a confidence interval of the average with approximation to the normal distribution. The confidence interval (CI) was set at 95%. For the cases of non-normal distribution, the median and its confidence interval were estimated^9^. For data following the normal distribution, the Pearson and non-normal correlation of Spearman’s were calculated, and the null hypothesis of null correlations was tested at the 0.05 level of significance (p<0.05). The notation (a, b, c, d) was used to characterize the averages for the statistical differences they presented among themselves, at a significance level of 0.05. Values accompanied by different letters in the same column in the tables differ and values accompanied by the same letter do not present statistical difference.

## RESULTS

### Characteristics of selected studies

A total of 24,818 patients were surveyed in 116 studies, of which 65 (56.03%) were prospective and 51 (43.96%) retrospective. Those submitted to RYGB were 6.630 (26.71%); SG 5.314 (21.41%); AGB 10.696 (43.09%) and BPD 2.178 (8.77%). As for the gender, there were 5.762 men (23.22%) and 19.056 women (76.78%).

The average age was 41.65 years (40.2-42.2) and BMI 46.78 kg/m^2^ (45.6-48). DM2 was reported in 71 studies 26.7% (22.7-30.0); dyslipidemia in 43, 38.85% (31,60-46,11); SA in 62, 45.69% (39.96-51.42) and OSA in 39 studies, 21.80% (16.70-41.30).

### Complications and postoperative mortality in the first 30 days

The bleeding rate in 74 studies was 0.6% (0.0-0.9); leaks 1.3% (0.7-1.8, 82 studies) and death 0.9% (0.3-1.8, 75 studies).

### Loss and regained overweight

Loss and regained overweight was of 21.29% (from 17.09 to 25.48) of excess weight loss in one month; 42.74% (39.14-46.34) in six months; 55.50% (49.00-63.50) in one year; 59.74% (55.49-64.00) in two years; 64.73% (59.94-69.51) in three years; 65.58% (56.97-74.19) in four years; and 63.86% (56.72-70.99) in five years.

### Resolution of comorbidities

The resolution rate of DM2 was 76.9% (69.0-82.0) after the operation; of dyslipidemia 74.0% (47.8-83.0); of SAH 61.80% (52.35-71.25) and AOS 75.0% (50.0-93.5).

### Individualized results for each technique

The average age of patients submitted to AGB was 43.18 years, BMI 43.91 kg/m^2^ and of women 79.44%. Of those submitted to SG, the average age was 41.90 years, BMI 49.47 kg/m^2^ and women 66.08%. In RYGB, the average age was 41.44 years, BMI 46.99 kg/m^2^ and female 79.37%. In BPD, average age was 41.73 years, BMI 55.18 kg/m^2^ and women 74.86%.

Of the patients submitted to AGB, 29.35% had DM2, 43.56% dyslipidemia, 16.60% apnea and 48.02% hypertension. Of those submitted to SG, 26.80% presented DM2, 31.12% dyslipidemia, 33.57% apnea and 43.78% hypertension. Under RYGB, 34.41% presented DM2, 47.85% dyslipidemia, 28.79% apnea and 44.62% hypertension. Undergoing BPD, 29.65% had DM2, 30.18% dyslipidemia, 34.05% apnea and 43.70% hypertension.

### Complications and postoperative mortality in the first 30 days

Of the patients submitted to AGB, 0.44% presented bleeding, 0.68% leaks and 0.05% death. Of those submitted to SG, 1.29% presented bleeding, 1.93% leaks and 0.16% died. Of those submitted to RYGB, 0.81% presented bleeding, 2.18% leaks and 0.60% died. Of those submitted to BPD, 2.09% bleeding, 5.23% leaks and 2.52% death were observed (Table 1).

**TABLE 1:** Complications (bleeding, leaks and death) by surgical technique

	% Bleeding	% Leaks	% Death
AGB	0.44 b	0.68 b	0.05 c
SG	1.29 b	1.93 a	0.16 bc
RYGB	0.81 b	2.18 a	0.60 b
BPD	2.09 a	5.23 a	2.52 a
p-value	0.0379	0.0097	0.0014

Averages followed by lower case distinct letters in the column differ from each other (Kruskal-Wallis 0.05 significance); AGB=adjustable gastric band; SG=vertical gastrectomy; RYGB=Roux-en-Y gastric bypass; BPD=biliopancreatic derivation

### Loss and regain of overweight

The loss of excess weight in patients who underwent AGB in the first six months was 27.05%; in the first year, 40.72%; in two years, 46.27%; in three years, 54.24%; in four years, 52.75%; and in five years, 48.35%. Therefore, the largest regain of weight was from the fourth year. In SG, in the first six months, it was 45.74%; in the first year, 55.17%; in two years, 59.18%; in three years, 68.85%; in five years, 52.7% - which characterizes the regain of weight after the fourth year. RYGB in the first six months was of 51.60% in the first year; of 64.58% in two years; 69.4% in three years; 70.22% in four years, 67.10%; and in five years, 71.4% - thus not showing any re-growth in the first five years. BPD in the first six months was 39.7%; in the first year, 61.47%; in two years, 66.08%; in three years, 66.78%; in four years, 75.5%; and, in five years, 77.9% - thus not showing any re-growth in the first five years (Table 2).

**TABLE 2:** Averages of the percentage of loss of overweight by time between surgical techniques, including the presumptions

Technique	^1^% EWL 6 months	% EWL 1 year	% EWL 2 years	% EWL 3 years	% EWL 4 years	% EWL 5 years
AGB	^2^27.05 b	40.72 b	46.27 b	54.24 b	52.75 b	48.35 b
SG	45.74 a	55.17 a	59.18 a	68.85 a	-	52.7 b
RYGB	51.60 a	64.58 a	69.4 a	70.24 a	67.10 a	71.04 a
BPD	39.70 a	61.47 a	66.08 a	66.78 a	75.50 a	77.90 a
pp	<0.001	<0.001	<0.001	0.0410	0.0199	0.0019

^1^% EWL=percentage of the loss of overweight; ^2^=averages followed by lower case distinct letters in the column differ from each other (Duncan’s test 0.05 significance); AGB=adjustable gastric band; SG=vertical gastrectomy; RYGB=Roux-en-Y gastric bypass; BPD=biliopancreatic derivation

### Resolution of comorbidities

Of the patients who underwent AGB, 46.8% presented resolution of DM2; 51.28% resolution of dyslipidemia; 54.5% of HAS; and 56.85% of OSA. Of those who underwent SG, 79.38% presented resolution of DM2; 58% dyslipidemia; 52.27% of HAS; and 51.43% of OSA. Of those who underwent RYGB, 79.86% presented resolution of DM2; 73.28% of dyslipidemia; 68.11% of HAS. As for OSA, 80.31% presented resolution. Of the patients who underwent BPD 90.78% presented resolution of DM2; 90.75% of dyslipidemia; 82.12% of HAS; and 92.5% of OSA (Table 3).

**TABLE 3:** Resolution of comorbidities according to surgical techniques

	% Diabete	% Dyslipidemia	% Apnea	% Hypertension
AGB	46.80 b	51.28 a	56.85 a	54.50 a
SG	79.38 a	58.00 a	51.43 a	52.27 a
RYGB	79.86 a	73.28 a	80.31 a	68.11 a
BPD	90.78 a	90.75 a	92.50 a	82.12 a
p-value	0.0058	0.1443	0.1112	0.1697

Means followed by different letters in the lower column is differences (Duncan’s test 0.05 significance); AGB=adjustable gastric band; SG=vertical gastrectomy; RYGB=Roux-en-Y gastric bypass; BPD=biliopancreatic derivation

## DISCUSSION

In the present day, it is observed that bariatric surgery is a good therapeutic modality for the treatment of morbid obesity^4^. In several prominent studies, such as those of Buchwald^3^, Schauer^20^ and Sugerman^22^, it can be noted that the improvement of comorbidities, together with the loss of excess weight, achieves good results with it.

As with many studies in the literature, it also sought to contribute to the understanding of the results of bariatric surgery. Taking into account that different techniques have been applied for more than two decades, it is important to analyze them in an individualized and comparative way, since, by offering different risks and results, even if tenuous, the different techniques show details that differentiate them and, knowing them better, one can think about its selective application for the benefit of the patients.

### Uniformity of patients

When comparing the groups of patients that compose the different techniques analyzed, it was observed that there was no statistical difference regarding the age averages. Also, no significant difference was observed when analyzing gender distribution, since women predominated in all groups, and only SG presented statistical difference in the other groups. Regarding the BMI, the patients of the SG and RYGB techniques did not present any statistical difference between them; the AGB presented lower average and those of BPD higher average than those of the others, which may have influenced in superior results of loss of excess weight for this technique. Therefore, we observed a uniformity of study in relation to age and distribution of genders, and in relation to BMI.

Regarding the distribution of comorbidities (SAH, DM2, OSA and dyslipidemia) in the preoperative period, there was no statistical difference between the patients in any of the techniques, thus demonstrating uniformity between the groups that were analyzed.

It has also been presented by other authors that the so-called dys-absorbing techniques present better results in the resolution of comorbidities and also in the loss of excess weight. In addition, it is observed that the more disabsorptive the technique applied is the better the resolution of comorbidities and sustained weight loss^3^, and this was also confirmed in this study.

On the other hand, purely restrictive techniques presented a lower risk of complications and mortality. In addition, it is easier to perform which is why vertical gastrectomy was proposed as the first approach for the duodenal switch operation to facilitate the operation of obese patients at high surgical risk^8^.

AGB is a purely restrictive method in which a silicone prosthesis is used in the surgical technique. In this study, this technique had low complication rates in the first 30 days; however, it should be noted that studies with larger follow-ups have shown complications of erosion and prosthesis migration in late postoperative periods^24^. In the current study, patients submitted to AGB presented a more modest weight loss and resolution of DM2 when compared to the other methods. Jan *et al*.^13^ demonstrated that patients submitted to it had initial weight loss results lower than those submitted to RYGB. These same authors also considered that the AGB has technical facility when compared with the other modalities^12^. Kim et al*.* ^14^ have reported that AGB should be considered in high-risk patients because it presents satisfactory results with acceptable risks.

SG is a purely restrictive method that does not use any type of prosthesis in its execution. Apparently, it is a technique that is easier to perform when compared to RYGB and BPD, since it does not require anastomosis. In it the gastric fundus is resected, and, according to Langer,^15^ this results in a decrease in the stimulus at the center of the hunger. In this study, SG presented a lower rate of death and leaks when compared to the disabsorptive techniques, but it is worth mentioning that the leaks of the proximal portion of the SG, when present, is difficult to solve^11^.

RYGB is a mixed technique (restrictive and disabsorptive) that presents good results in the treatment of comorbidities and also in the loss of excess weight^19^. Maher et al.^17^ reported that it should be the one of choice in bariatric surgery, mainly in super obese and diabetic patients. In this study, BPD presented the best results in relation to weight loss and resolution of DM, but showed higher complication rates than purely restrictive techniques, although lower than biliopancreatic derivation procedures.

BPD, represented by the operations of “Scopinaro” and “duodenal switch”, is, in some cases, the surgical modality chosen for super obese, since, according to Hess^7^, there is the option to perform SG as the first surgical approach of the duodenal switch with potential definitive approach. And, according to Sucandy^21^, patients with BMI above 50 kg/m^2^ are more likely to remain with BMI below 35 kg/m^2^ when subjected to BPD than by purely restrictive methods. However, it is worth mentioning that this surgical modality causes important nutritional disorders of vitamins and minerals, a factor that was not analyzed in the present study. Homan^10^ describes that, even with postoperative supplementation, most patients presented high rates of malnutrition. Nelson^16^ demonstrated that BPD promotes longer operative time and a higher conversion rate for laparotomy when compared to DGYR. Although malnutrition, surgical approach and conversion rate for laparotomy were not analyzed in this study, these factors may justify BPD being the least casuistic operation among the techniques studied here. In this study, BPD was the operative modality that demonstrated better resolution of comorbidities, as well as loss of excess weight; however, it was the technique of higher complication rates in the first 30 days, so its indication seems to be very restricted.

### Limitations of the study

This study presents limits that must be emphasized, such as the fact of presenting works with different statistical forces for the same analysis. In addition, studies extracted from the literature show the same surgical technique applied by different professionals, in places with different operative infrastructure, therefore, it is a heterogeneous study. Another relevant limit is due to the fact that it does not incorporate the rate of malnutrition and consequent quality of life in the different techniques, since the research incorporated here does not describe these variables. This may explain why the BPD technique has the best results of weight loss and resolution of comorbidities, and yet be the least casuistic

## CONCLUSIONS

AGB has the lowest morbidity and mortality and is the worst in EWL and resolution of DM2. SG has low morbidity and mortality, good resolution of comorbidities and EWL lower than RYGB and BPD. RYGB presents morbidity and mortality higher than AGB, good resolution of comorbidities and EWL similar to BPD. BPD is the worst in mortality and bleeding and better in EWL and resolution of comorbidities.
